# Facial expression recognition via variational inference

**DOI:** 10.1038/s41598-026-38734-x

**Published:** 2026-02-05

**Authors:** Gang Lv, JunLing Zhang, Chiki Tsoi

**Affiliations:** 1 Learning and Information Center, JinHua Open University, No. 18 Qingzhao Road, JinHua, 321000 Zhejiang China; 2 Advanced Institute of Information Technology, Peking University, No. 233 Yonghui Road, Xiaoshan District, Hangzhou, 311200 Zhejiang China

**Keywords:** Facial expression recognition, Variational inference, Probabilistic model, Feature representation, Computational biology and bioinformatics, Mathematics and computing

## Abstract

Facial expressions in the wild are rarely discrete; they often manifest as compound emotions or subtle variations that challenge the discriminative capabilities of conventional models. While psychological research suggests that expressions are often combinations of basic emotional units, most existing FER methods rely on deterministic point estimation, failing to model the intrinsic uncertainty and continuous nature of emotions. To address this, we propose POSTER-Var, a framework integrating a Variational Inference-based Classification Head (VICH). Unlike standard classifiers, VICH maps facial features into a probabilistic latent space via the reparameterization trick, enabling the model to learn the underlying distribution of expression intensities. Furthermore, we enhance feature representation by introducing layer embeddings and nonlinear transformations into the feature pyramid, facilitating the fusion of hierarchical semantic information. Extensive experiments on RAF-DB, AffectNet, and FER+ demonstrate that our method effectively handles fine-grained expression recognition, achieving state-of-the-art performance. The code has been open-sourced at: https://github.com/lg2578/poster-var.

## Introduction

Facial expressions are the manifestation of emotions on the face and are the primary form of emotional expression. Facial expression recognition (FER) holds vast research potential and application worth in human-computer interaction, psychology, intelligent robotics, intelligent surveillance, virtual reality and synthetic animation.

**Fig. 1:**
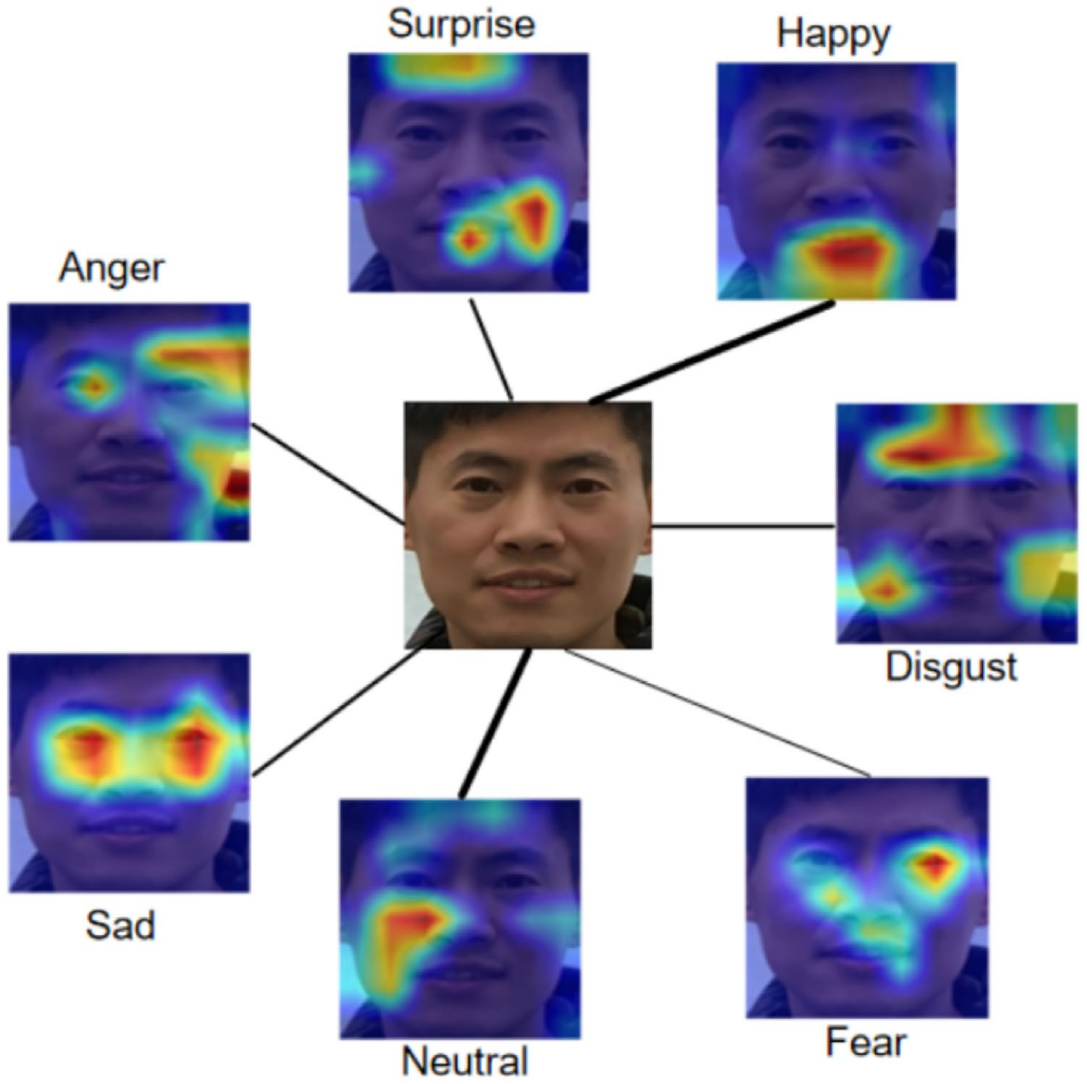
Mixed features that map to different expression classes coexisting in a facial image. Thicker connecting lines represent higher predicted probabilities for the corresponding class. Class Activation Maps (CAMs) are generated using Grad-CAM^14^, the heatmap shows which regions of the image contribute positively to a specific class, even if that class is not the model’s final prediction.

In recent years, with the continuous development of deep learning, facial expression recognition has achieved remarkable research progress^1–7^. However, existing FER literature predominantly discretizes and orthogonalizes emotional states. By relying on deterministic point estimation approaches for coarse classification, these methods fail to capture the high-dimensional and continuous spectrum of human emotion. FACS^8^ decomposes facial expressions into combinations of multiple action units (AUs), each AU corresponds to the movement of a specific facial muscle or group of muscles, and the same AU may occur across different expressions. Psychological studies^9^ and previous FER work^10,11^ have also shown that most emotions occur as combinations, mixtures, or compounds of the basic emotions, and multiple emotions always have different intensities within a single facial image, especially in the real world, as show in Fig. 1. Calibrate the feature distribution within a single image and making the final decision is crucial for improving recognition accuracy. Salient feature suppression^12^ encourages the model to focus on weaker features by suppressing dominant ones. LDL^13^ introduce a simple but efficient label distribution learning method as a novel training strategy and leverage depthwise convolution to capture local and global-salient facial features.

Inspired by variational autoencoder (VAE) module widely used in generative models, we propose a novel method that enables the model to better balance features corresponding to different expression classes. During training, the model performs reparameterization via the proposed Variational Inference-based Classification Head (VICH) to learn the underlying distribution of expression combinations. This method encourages the model to learn the probabilistic distribution of expression combinations. Heatmap visualizations demonstrate that the model is able to make decisions by considering broader regional features.

Variational Inference (VI)^15^ offers a principled framework for incorporating uncertainty into deep models. It is an approximation technique for Bayesian inference that transforms the problem of computing the intractable posterior distribution into an optimization task by approximating it with a simpler, tractable distribution. While VI has shown great success in generative modeling^16^, its application to classification tasks remains limited. We argue that previous methods typically decode the latent vector before feeding it into the classifier. For a pure classification task, this decoding step is redundant and compromises the model’s performance. Moreover, during inference, using the mean of the learned Gaussian distribution helps reduce the intrinsic variability of the features. So We introduced two improvements to the reparameterization process. First, sampling is applied only during training, while the learned distribution mean is output directly during inference. Second, the final fully connected classifier is removed, allowing the reparameterized output to serve directly as the prediction. Furthermore, we enhance multi-scale feature fusion by incorporating layer embedding and nonlinear transformation into the baseline fusion module. The layer embedding encodes the positional and semantic level of each feature map within the feature pyramid, allowing the model to better distinguish and integrate information from different scales. The nonlinear transformation enriches the representation capability of fused features, facilitating more effective learning of complex patterns.

Overall, our contributions are summarized as follows:

- We propose a novel Variational Inference-based Classification Head (VICH). VICH is designed to learn the underlying distribution of expression combinations, thereby encouraging the model to calibrate the feature distribution and to make decisions based on broader regional features.
- We enhance multi-stage feature fusion by incorporating layer embeddings and nonlinear transformations, which effectively harmonizes the semantic gaps between different levels and adaptively extracts task-relevant high-level abstractions within the feature pyramid.
- Our method outperforms current SOTA approaches across multiple Facial Expression Recognition (FER) benchmarks, achieving accuracies of 92.76% on RAF-DB, 67.91% on AffectNet (7 classes), 64.27% on AffectNet (8 classes), and 91.89% on FER+.

## Related work

### Facial expression recognition

With the continuous advancement of deep learning technologies, significant progress has been made in the research of facial expression recognition. MHCNN^3^ uses multi-task learning to automatically crop edge-free faces and recognize facial expressions, age, gender. TransFER^4^ combines multi attention dropping and multi-head self attention dropping mechanisms to learn rich relation-aware local representations. MTSD-CF^17^ uses a multi-task self-distillation method with coarse- and fine-grained labels, providing additional guidance for the extraction of discriminative features. QCS^1^ uses cross similarity attention and quadruplet cross similarity to adaptively mine discriminative features within the same class while simultaneously separating interfering features across different classes. ArcFace^2^ introduces an additive angular margin loss to further improve the discriminative power of the face recognition model and to stabilise the training process. POSTER^5^ combines pre-trained facial landmark detector^7^ with image features detector^2^ through a two-stream pyramidal cross-fusion transformer. POSTER++^6^ removes the image-to-landmark branch from the original two-stream design of POSTER, performs multi-scale feature extraction directly from the image backbone as well as from the facial landmark detector, it significantly reduces model parameters and computational cost while slightly improving model performance.

In summary, the aforementioned FER studies predominantly adopt deterministic point estimation approaches. However, these methods often struggle with the inherent ambiguity of facial expressions and the label noise present in large-scale datasets. By reducing a complex emotional state to a single hard label, deterministic models fail to capture the subtle transitions between different emotions and are sensitive to subjective annotation biases, which limits their robustness in real-world scenarios.

### Variational inference-based classification network

In machine learning, parameter estimation methods are generally categorized into point estimation and Bayesian inference. The former yields a single optimal parameter value, while the latter models parameters as probability distributions to capture uncertainty^15^. VI can be viewed as an approximate form of Bayesian inference, where the intractable posterior is replaced by a parameterized distribution.

Given the great success of the VI in generative tasks, some studies have also applied VI in classification tasks. AEVB^35^ uses an improved parameter reparameterization technique that leads to better performance of variational inference in classification tasks. AAE^18^ is a novel framework for speech emotion recognition that employs variational inference of latent variables and reconstruction of the speech signal. The VAE-based classifier^19^ removes the decoder and directly connects the latent variables to a data classifier to perform the learning task, aiming to jointly optimize the encoder and the classifier with end-to-end training. FRA^20^ is a face representation augmentation method, shifts its focus towards manipulating the face embeddings generated by any face representation learning algorithm to create new embeddings representing the same identity and facial emotion but with an altered posture.

The architectural designs of these VI-based approaches provide valuable insights for improving our POSTER-Var model. By eliminating both the decoder and the final fully connected (FC) classifier used in conventional VI-based classification models, we introduce a novel classification head that substantially improves model performance and streamlines the overall architecture.

### Attention mechanism

In deep learning, attention mechanisms often introduce element-wise multiplication as a core operation, allowing neural networks to dynamically emphasize or suppress different parts of the learned representation. For instance, in the Squeeze and Excitation block^21^, the output of the excitation module is multiplied with the original feature map to reweight channels according to their relative importance. Similarly, CBAM^22^ applies both channel and spatial attention maps via multiplicative scaling, thereby enabling the model to focus on salient information from multiple perspectives. ViT^23^ treats an input image as a sequence of fixed-size patches and uses a dot-product self-attention mechanism to compute weighted outputs. Micro_NesT^24^ uses a shallow feature extraction module and a hierarchical attention extraction module, enabling information interaction between different patches through aggregation modules. MFD^25,26^ is proposed to integrate features in the whole training set by memory-attention layers, which encourages the heterogeneous features with the same identity to present higher similarity.

Taken together, fusing multiple attention mechanisms allows the model to capture multi-scale and multi-dimensional features, enhancing representational capacity and generalization. In our proposed method, four different attention mechanisms are effectively integrated to enhance model performance.

## Method

### Baseline

We adopt POSTER++ as the baseline, as it significantly reduces the model parameters and computational cost while achieving slightly better performance than POSTER. POSTER++ employs IR50^2^ as an image backbone to extract image features at three different scales, while MobileFaceNe^7^ is used to obtain the landmark features at the corresponding scales.

Let the input image ${\textbf{X}} \in \mathbb {R} {^{3 \times h \times w}}$, where 3 denotes the number of channels, *h* and *w* are the height and width of the image. In baseline, the image features ${{\textbf{X}}_{{\text {img}}}} \in \mathbb {R}{^{c \times h \times w}}$ as well as the landmark features ${{\textbf{X}}_{{\text {lm}}}} \in \mathbb {R}{^{c \times h \times w}}$ are fused using global context window-based cross-attention^27^, and then concatenated along the channel dimension. The fused features ${{\textbf{X}}_{{\text {fusion}}}} \in \mathbb {R}{^{n \times d}}$ are subsequently processed by a lightweight two-layer ViT to capture long-range dependencies, followed by a feed-forward network for classification.

### Architecture

**Fig. 2:**
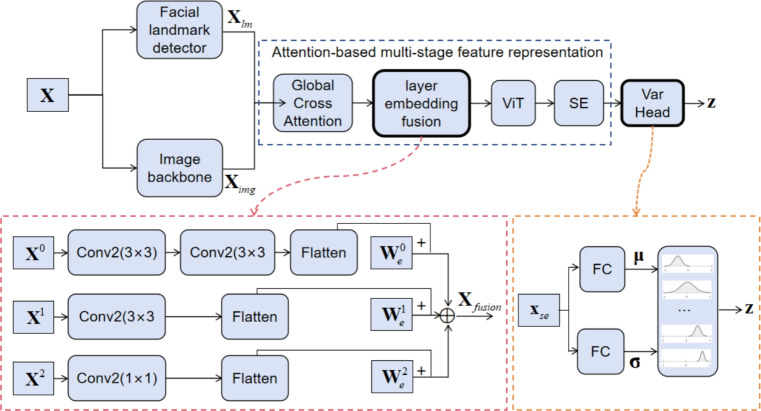
Our proposed POSTER-Var architecture for FER.

We propose POSTER-Var, which extends baseline from two pivotal perspectives. Firstly, we introduce a layer-embeding feature fusion module. Secondly, we design a classification head based on variational inference. Unlike previous studies that feed either the reconstructed output or the latent variables into a separate classifier, our method directly treats the reparameterized representations as the final classification outputs during training. As illustrated in Fig. 2, the components highlighted with bold lines represent the improvements introduced over the baseline model.

A detailed explanation of the figure can be found in the following subsection. Compared with the baseline, the learnable positional embedding $\textbf{W}_e$ has a size of only $3 \times 768$, and the VICH module is only $2 \times 7 \times 768$. Despite the negligible increase in model size and computational cost, these components effectively improve the model’s performance.

### Attention-based multi-stage feature representation

In POSTER-Var, various attention mechanisms are employed. Features from different feature extractors are first fused using global cross-attention:

1 $$\begin{aligned} {\mathop {\text {Attention}}\nolimits } \left( {{\textbf{Q}}_l^*,{{\textbf{K}}_l},{{\textbf{V}}_l}} \right) = softmax\left( {\frac{{{\textbf{Q}}_l^*{\textbf{K}}_l^T}}{{\sqrt{{d_k}} }}} \right) {{\textbf{V}}_l} \end{aligned}$$

Here,subscript $l \in \{ 0,1,2\}$ denotes different feature layers. $\textbf{K}_l$ and $\textbf{V}_l$ are generated by applying a linear projection with learnable weights to the image features ${\textbf{X}_{\text {img}}^l}$. In contrast to the standard self-attention mechanism, in our method the ${\textbf{Q}_l^*}$ is obtained by reshaping the landmark features ${\textbf{X}_{\text {lm}}^l}$ without applying a learnable linear projection:

2 $$\begin{aligned} \begin{aligned} \textbf{Q}_l^*&= \operatorname {reshape}\left( \textbf{X}_{\text {lm}}^{l} \right) \\ \textbf{K}_l&= \textbf{X}_{\text {img}}^{l} W_{k_l} \\ \textbf{V}_l&= \textbf{X}_{\text {img}}^{l} W_{v_l} \end{aligned} \end{aligned}$$

In the second stage, the model adds the input embeddings with the layer positional embedding vector using broadcasting, to incorporate sequential positional information:

3 $$\begin{aligned} \begin{aligned}&\widetilde{X^l} = \operatorname {Flatten}\left( \operatorname {Embed}^l(\textbf{X}^l) \right) + W_e^l \\&X_{\text {fusion}} = \operatorname {concat}\left( \widetilde{X^0}, \widetilde{X^1}, \widetilde{X^2} \right) \end{aligned} \end{aligned}$$

$W_e^l$ is the learnable layer positional embedding, $\operatorname {Embed}^l$ refers to the corresponding embedding layer, which applies different convolution operations to normalize different layers. In the third stage, ${{\textbf{X}}_{{\text {fusion}}}} \in \mathbb {R}{^{n \times d}}$ is further processed by a 2 layers ViT to model global contextual relationships and get the representation vector ${{\textbf{x}}_{repr}} \in \mathbb {R} {^d}$. In the fourth stage, $\textbf{x}_{repr}$is then refined via an enhanced Squeeze-and-Excitation (SE) module to adaptively recalibrate and enhance informative feature channels:

4 $$\begin{aligned} \textbf{x}_{se} = \operatorname {SE}\_{\text {block}}(\textbf{x}_{repr}) = \textbf{x}_{repr} \odot \sigma \Big ( W_2 \cdot \operatorname {ReLU}( W_1 \cdot \textbf{x}_{repr} ) \Big ) \end{aligned}$$

$\odot$ denotes element-wise multiplication, $\sigma (\cdot )$ denotes the Sigmoid activation function, $\operatorname {ReLU}(\cdot )$ denotes the Rectified Linear Unit activation function; $W_1,W_2 \in \mathbb {R}^{d \times d}$ are the weight matrices of the two fully connected layers.

### VI-based classifier

The VI module incorporates the reparameterization trick, is a technique commonly employed in generative models to sample latent variables from a learned distribution. In contrast, we repurpose this mechanism for classification tasks, allowing probabilistic reasoning and uncertainty quantification in the decision process. During the training phase, the module samples from a Gaussian distribution parameterized by the predicted mean and log-variance, introducing stochasticity while preserving gradient flow through the sampling process.

5 $$\begin{aligned} \textbf{z} = \boldsymbol{\mu } + \boldsymbol{\varepsilon } \cdot \exp \!\left( \frac{\log \boldsymbol{\sigma }^{2}}{2} \right) \end{aligned}$$

Here,$\boldsymbol{\varepsilon } \sim \mathcal {N}(0,I)$ denotes random noise sampled from the standard multivariate normal distribution , $\boldsymbol{\mu }$ and $\boldsymbol{\sigma }$ are learnable vectors generated by the encoder network. $\boldsymbol{\mu }$ represents the mean of the approximate posterior distribution $q(\textbf{z} \mid \textbf{x})$, indicating the central location of the latent variable $\textbf{z}$ conditioned on the input $\textbf{x}$. $\boldsymbol{\sigma }$ represents the standard deviation of this distribution, capturing the uncertainty or spread around the mean. These parameters are used to define a diagonal Gaussian distribution in the latent space, from which $\textbf{z}$ is sampled using the reparameterization trick.

In the testing phase, to ensure stable and deterministic predictions, the module bypasses sampling and directly outputs the mean as the final latent representation for classification.This is the key difference between our method and previous classification approaches based on VI.

## Experiments

### Datasets

We verify the effectiveness of POSTER-Var on several FER benchmarks, such as RAF-DB^28^, AffectNet^29^ and FER+^30^.

**RAF-DB**. Real-world Affective Faces Datasets(RAF-DB)^28^, developed by Beijing University of Posts and Telecommunications, comprises approximately 30,000 facial images collected from thousands of individuals in unconstrained environments. In this study, we utilized the RAF-DB Basic Emotion Subset, a widely adopted benchmark dataset consisting of 15,339 real-world facial images, each annotated with one of seven basic emotion classes: Happy, Sad, Surprise, Anger, Disgust, Fear, and Neutral. To ensure annotation consistency and reliability, each image was labeled by approximately 40 independent raters, and the final label was derived using the Expectation-Maximization (EM) algorithm. According to the standard partition, the dataset is divided into 12,271 training images and 3,068 test images, making it well-suited for training and evaluating facial expression recognition models.

**AffectNet**. AffectNet^29^ developed by University of Denver, is currently the largest publicly available dataset in the field of FER, containing approximately 1 million facial images associated with emotion labels. The dataset primarily includes 8 classes of basic emotions: Neutral, Happy, Anger, Sadness, Fear, Surprise, Disgust, and Contempt. In addition to these annotated classes, AffectNet also includes three extra labels: None for faces that do not express any recognizable emotion, Uncertain for ambiguous expressions that annotators could not confidently classify, and No-face for images where no face was detected. To ensure the quality and reliability of model training, we mainly use the 7-class version of AffectNet (excluding Contempt) and the 8-class version in this study. AffectNet (7 cls) consists of 283,902 training images and 3,500 validation images (500 images per category). AffectNet (8 cls) consists of 287,652 training images and 4,000 validation images (500 images per category).

**FER+**. FER+^30^ developed by Microsoft Research, is an enhanced version of the original FER2013 dataset,it contains 28,709 training, 3,589 validation, and 3,589 test images. In FER+, each image has been labeled by 10 crowd-sourced taggers, which provide better quality ground truth for still image emotion than the original FER labels. Having 10 taggers for each image enables researchers to estimate an emotion probability distribution per face. This allows constructing algorithms that produce statistical distributions or multi-label outputs instead of the conventional single-label output. Folllowing^1,30^, we utilized FER+ to filter out samples labeled as ’no face’ or ’unknown’ and reported the overall accuracy on the test set.

### Experiment details

Training is conducted for 200 epochs using the AdamW optimizer^31^ to ensure robust generalization and stable convergence. Beyond standard data augmentations like random horizontal flipping and random erasing, the optimization process on RAF-DB, AffectNet, and FER+ is supervised by a joint loss function that leverages both Cross-Entropy (CE) and Kullback-Leibler (KL) divergence. All experiments were conducted on a single NVIDIA RTX 3090 via PyTorch 2.5. To ensure the comparability of results, all methods were trained under identical conditions. The detailed training configurations and hyperparameters are provided in Table 1.

**Table 1:** Training configurations.

Configs	RAF-DB	AffectNet	FER+
Optimizer	AdamW	AdamW	AdamW
Init LR	9e-6	2e-5	3e-5
Weight Decay	1e-4	1e-4	1e-4
Batch Size	48	48	48
Max Epochs	250	200	200
LR Schedule	Exp. ($\gamma =0.98$)	Exp. ($\gamma =0.90$)	Exp. ($\gamma =0.96$)
Augmentation	Resize: $224^2$	Resize: $236^2$	Resize: $232^2$
	H. Flip	H. Flip	H. Flip
		Rot. ($12^\circ$)	Rot. ($10^\circ$)
		Random Crop ($224^2$)	Random Crop ($224^2$)
	Color Jitter (0.2)	Color Jitter (0.2)	Color Jitter (0.2)
	Normalize()	Normalize()	Normalize()
	Random Erasing	Random Erasing	Random Erasing
Classes	7	7/8	8
Loss Function	CE + $\lambda$ KL	CE + $\lambda$ KL	CE + $\lambda$ KL

Table 2 presents the performance comparison between our method and recent advanced approaches in the field of emotion recognition. Overall, emotion recognition techniques demonstrate continuous performance improvement across multiple benchmark datasets. POSTER-Var achieves state-of-the-art (SOTA) performance across several benchmarks, with accuracies of 92.76% on RAF-DB, 67.91% on AffectNet (7 classes), and 91.89% on FER+. These results consistently surpass the leading DCS method, which achieves 92.57%, 67.66%, and 91.41% respectively. The model also achieves a competitive 64.27% accuracy on the 8-class AffectNet, aligning with top-tier SOTA results. These results underscore the model’s exceptional capability in characterizing complex facial expressions. Such gains are primarily attributed to our probabilistic modeling of expression variation, which empowers the framework to effectively capture nuanced, subject-specific differences.

**Table 2:** Comparison with SOTA methods.

Methods	Year	RAF-DB	AffectNet (7 cls)	AffectNet (8 cls)	FER+
PSR^32^	CVPR 2020	88.98	63.77	60.68	89.75
EfficientFace^13^	AAAI 2021	88.36	63.70	60.23	–
Meta-Face2Exp^33^	CVPR 2022	88.54	64.23	–	–
POSTER^5^	ICCV 2023	92.05	67.31	63.34	91.62
MFER^34^	T-AFFC 2024	92.08	67.06	63.15	91.09
POSTER++^6^	PR 2025	92.21	67.49	63.77	–
DCS^1^	AAAI 2025	92.57	67.66	**64.40**	91.41
MTSD-CF^17^	ESWA 2025	92.63	66.26	–	–
Ours$^{*}$	2026	**92.76**	**67.91**	64.27	**91.89**

*Detailed training logs and reproducibility results are available at: https://swanlab.cn/@lezi.

Bold values indicate the best performance.

### Ablation study

To evaluate the effectiveness of the proposed layer embedding and VICH module, we conduct extensive ablation studies on three benchmark facial expression recognition datasets: RAF-DB, AffectNet (7 and 8 classes), and FER+. The results are summarized in Table 3. Inference time is calculated as the average of 1000 runs on a single NVIDIA 3090 GPU. Full POSTER-Var Model achieves the best results across all datasets, RAF-DB: 92.76%, AffectNet (7 cls): 67.91%, AffectNet (8 cls): 64.27%, FER+: 91.89% with negligible computational overhead, maintaining an inference time nearly identical to the baseline.

**Table 3:** Ablation results of POSTER-Var.

Methods	RAF-DB	AffectNet (7 cls)	AffectNet (8 cls)	FER+	Inf. Time (ms)
Ours	**92.76**	**67.91**	**64.27**	**91.89**	1.502
w/o Layer Emb.	92.66	67.85	64.24	91.85	1.502
w/o VI Module	92.50	67.66	64.02	91.69	1.492
Baseline	92.21	67.49	63.77	91.62	1.491

Bold values indicate the best performance.

${\textbf {Layer embedding}}$. Removing the layer positional embedding leads to a consistent performance drop. On RAF-DB, accuracy decreases slightly to 92.66%. On AffectNet (7 cls) and (8 cls), accuracies drop to 67.85% and 64.24%, respectively. On FER+, accuracy decreases slightly to 91.85%. This suggests that the layer embedding helps improve the model’s capacity to capture hierarchical feature representations.

${\textbf {VICH module}}$. Disabling the VICH module results in a more significant performance decline. RAF-DB drops to 92.50%, and AffectNet (7 cls) and (8 cls) decline to 67.66% and 64.02%, on FER+ accuracy falls to 91.69%. This indicates that the VICH module plays a vital role in modeling uncertainty and enhancing generalization, especially on more complex datasets like AffectNet and FER+.

Both the layer embedding and VICH module are crucial to the success of POSTER-Var. Their removal consistently degrades performance, confirming their complementary contributions to improving expression recognition accuracy. Notably, the VICH module appears slightly more impactful, particularly in datasets with greater variation and class imbalance like AffectNet.

### Visualization

**Fig. 3:**
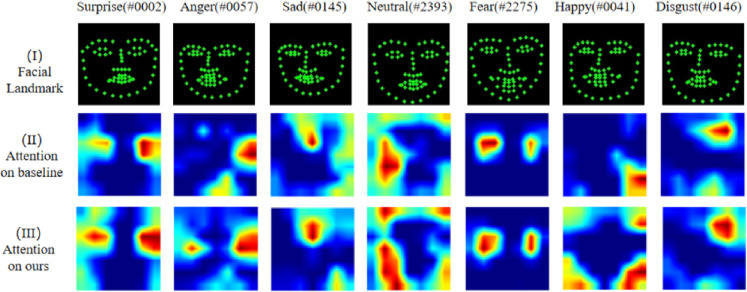
Attention visualization on facial images of different classes. Recognisable faces in the figure have been replaced by their dataset indices to comply with privacy policies, label #xxxx denotes the image indexed xxxx in the RAF-DB test set.

We conducted a visual analysis comparing the baseline and POSTER-Var(ours) on RAF-DB. Figure 3 shows attention visualization on facial images of different classes, include visualized facial landmarks and class activation maps. We can see that both models focus on similar regions, indicating that they are both able to learn the key features. However, the activation regions produced by POSTER-Var are more extensive and better aligned with key facial landmarks than those of the baseline. This broader attention helps the model capture the uncertainty of facial expressions and make decisions based on more comprehensive regional features and reducing the likelihood of misclassification.

**Fig. 4:**
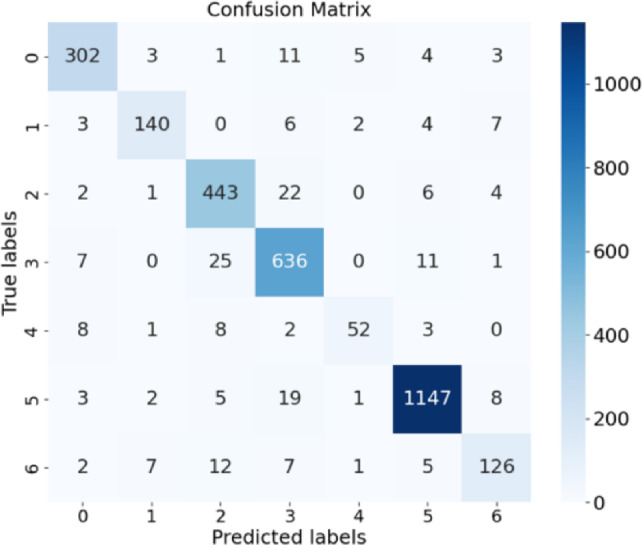
Confusion matrix of ours method on RAF-DB.

The more detailed experimental results of POSTER-Var on RAF-DB are presented in Table 4 and Fig. 4 The class distributions in the training and validation sets of RAF-DB are relatively consistent, and the classification performance of individual classes tends to correlate with the number of training samples. Nevertheless, our model still achieves satisfactory precision for classes with fewer samples, such as sad, fear, and neutral.

**Table 4:** Sample distribution and performance per expression Class.

	Suprise	Anger	Sad	Neutral	Fear	Happy	Disgust
Training samples	1290	705	1982	2524	281	4772	717
Testing samples	329	162	478	680	74	1185	160
Recall	91.79%	86.42%	92.68%	93.53%	70.27%	96.79%	78.75%
Precision	92.35%	90.91%	89.68%	90.47%	85.25%	97.20%	84.56%

From Fig. 4, we observe that the neutral class(label=3) exhibits a significantly higher false positive rate compared to the happy class(label=5). The neutral class has 70 false positives, far exceeding the 38 of the happy class, resulting in a considerably higher false positive rate (9.92% vs. 3.21%). This suggests that the model is more prone to misclassify other emotions as neutral. However, the neutral class contains only about half as many training samples as the happy class, indicating that this phenomenon is not due to class imbalance.

**Fig. 5:**
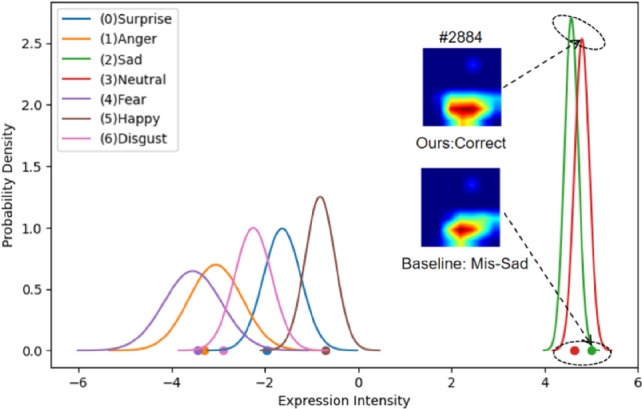
Normal distributions of seven emotions learned by VICH for a given image. Points and solid curves denote the outputs of the baseline and POSTER-Var, respectively. The final prediction is determined by the expression category with the highest intensity value. Recognisable faces in the figure have been replaced by their dataset indices to comply with privacy policies, label #xxxx denotes the image indexed xxxx in the RAF-DB test set.

Benefiting from the ability of the VICH module to learn the underlying distribution of expression combinations, we can easily plot the expression feature distribution of a given image, as shown in Fig. 5. The x-axis represents the expression intensity predicted by the model, and the class with the highest intensity among the seven categories is taken as the final classification result. The baseline output (indicated at the origin) incorrectly classifies the image as sad instead of neutral. In contrast, our model produces the correct classification. The reparameterization strategy employed during training encourages the model to evaluate images across a broader range of intensity values, strengthens the calibration of expression features, and enlarges inter-class discriminative distances.

## Conclusions

In this paper, we addressed the limitation of deterministic point estimation in capturing the complexity of real-world facial expressions. By acknowledging that expressions are often combinations of basic emotions, we proposed POSTER-Var, incorporating a VI-based Classification Head. This approach fundamentally shifts the learning paradigm from fitting specific points to modeling feature distributions, thereby quantifying the uncertainty inherent in compound expressions. Coupled with our enhanced multi-scale feature fusion, the proposed method achieves superior performance on benchmark datasets. Our work suggests that probabilistic modeling is a promising direction for the next generation of fine-grained and robust Affective Computing systems. Future research will focus on integrating Domain Generalization (DG) frameworks with our variational architecture. Specifically, we aim to explore disentangled representation learning to effectively separate emotion-specific latent variables from identity-related nuisance factors. This will ensure that the learned feature distributions are more invariant across different datasets, ultimately facilitating the deployment of POSTER-Var in diverse, real-world human-computer interaction applications.

## Data Availability

The RAF-DB dataset is available from the original authors upon request for non-commercial research purposes. Researchers affiliated with academic institutions may request access by contacting the authors as described at http://whdeng.cn/RAF/model1.html. The FER+ dataset is available at https://github.com/microsoft/FERPlus. The AffectNet dataset can be requested from the original authors at https://mohammadmahoor.com/pages/databases/affectnet/ by eligible researchers (e.g., Principal Investigators) subject to a signed license agreement.
